# Ce–Zr-based mixed oxide catalyst for oxidative depolymerization of kenaf stalk (biomass) into vanillin

**DOI:** 10.1186/s40643-023-00698-5

**Published:** 2023-11-07

**Authors:** Hifza Rouf, Anita Ramli, Nur Akila Syakida Idayu Khairul Anuar, Normawati Mohamad Yunus

**Affiliations:** 1 HICoE Centre of Biofuels and Biochemicals Research, Universiti Teknologi PETRONAS, 32610 Seri Iskandar, Perak Malaysia; 2 Fundamental and Applied Sciences Department, Universiti Teknologi PETRONAS, 32610 Seri Iskandar, Perak Malaysia; 3 Centre of Research in Ionic Liquids (CORIL), Institute of Contaminant Management for Oil and Gas, Department of Fundamental and Applied Sciences, Universiti Teknologi PETRONAS, 32610 Seri Iskandar, Malaysia

**Keywords:** Biomass, Vanillin, Oxidative depolymerization, Citrate complexation method, CeZrO_2_–CA

## Abstract

Since petroleum became depleted, rapid attention has been devoted to renewable energy sources such as lignocellulosic biomass to produce useful chemicals for industry (for instance vanillin). Three primary components of lignocellulose are lignin, cellulose, and hemicellulose. This paper uses microwave-assisted technology to oxidize the kenaf stalk (lignocellulosic biomass) and extract lignin to produce vanillin. Catalysts with variable acid–base and redox properties are essential for the mentioned effective conversion, for this reason, CeO_2_–CA, ZrO_2_–CA, and CeZrO_2_–CA catalysts were synthesized. The citrate complexation method was used for the catalyst synthesis and the physicochemical characteristics were analyzed by XRD, FTIR, FE–SEM, TEM, BET, and TPO. The characterization results demonstrated that CeZrO_2_–CA shows the smallest sized crystallites with a large specific surface area among the other chosen catalysts. For vanillin production, the effect of reaction temperature, reaction time, and catalyst loading was studied. It was observed that compared to other catalysts, CeZrO_2_–CA produced the highest vanillin yield of 9.90% for kenaf stalk for 5 wt% of CeZrO_2_–CA at 160 °C for 30 min. Furthermore, vanillin production using extracted lignin is studied keeping CeZrO_2_–CA as a catalyst and with the same operating parameters, which yielded 14.3% of vanillin. Afterward, the change in yield with respect to pH is also presented. Finally, the recyclability of catalyst is also studied, which showed that it has a strong metal support and greater stability which may give industrial applications a significant boost.

**Graphical Abstract:**

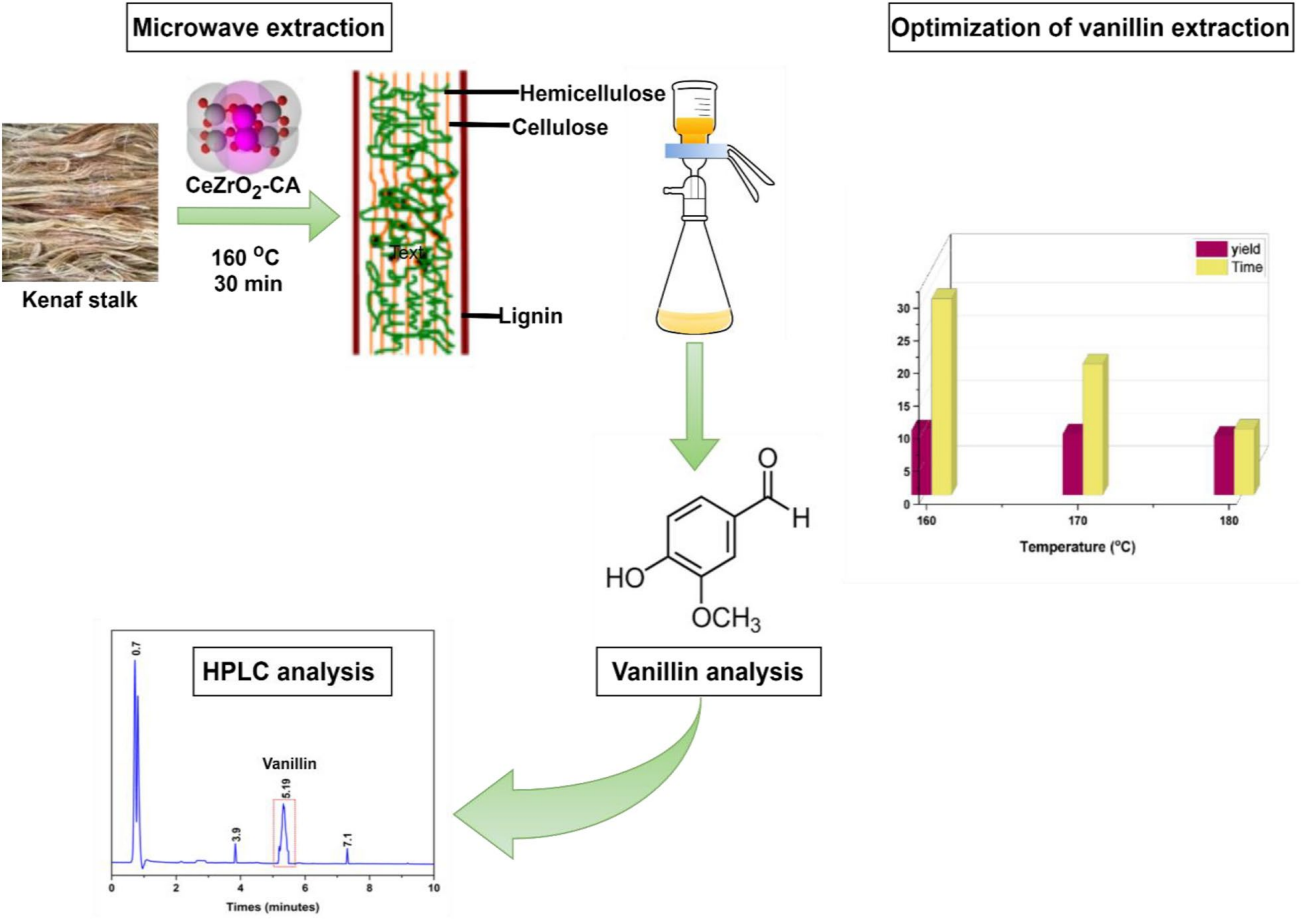

## Introduction

Vanillin is a molecule of interest because it can be utilized in various beneficial goods. As a result, the market demand for it has been increasing daily. Due to the number of challenges, such as small cultivation areas with a suitable environment and lengthy and laborious methods, the production of natural vanillin from vanilla orchid pods is minimal. Roughly 20,000 tons of vanillin is produced each year (Bomgardner 2014), with lignin accounting for 15% of the total (around 3000 tons per year) (Silva et al. 2009). As a result, vanillin has the potential to become an essential renewable aromatic building component.

To reduce the dependency on petroleum refineries for the production of useful chemicals and fuels, the world is transitioning toward renewable resources, notably biomass conversion (Dessbesell et al. 2020; Haruna et al. 2022, 2023). Researchers widely study the depolymerization of lignocellulosic biomass into useful products (Lee et al. 2021; Baksi et al. 2023; Kong et al. 2023; Yang et al. 2023). Lignocellulose is mainly composed of cellulose, hemicellulose, and lignin. Lignin, a significant biopolymer derived from lignocellulosic biomass (accounts for 30% by weight), is a potential feedstock to produce green aromatic chemicals (Kong et al. 2023). Lignin possesses a heterogeneous structure containing aryl ether (i.e., composed of *β-o*-4 linkage) as the most abundant structural unit (up to 60% of all linkages), followed by *β*-5, *β–β*, and other minor units (Li et al. 2015; Vega-aguilar et al. 2021).

Because lignin's carbon–carbon bonds are so strong, breaking them during depolymerization can be difficult, which affects the lignin's reactivity and causes the product to vary depending on the depolymerization technique, lignin isolation procedure, and lignin source (Li et al. 2015; Li and Takkellapati 2018; Pérez et al. 2022). In previous reports, various chemical and thermal-based lignin depolymerization techniques have been developed and used (Liu et al. 2020; Schutyser et al. 2018) which offers several drawbacks, such as poor selectivity and more energy usage**.** While microwave-assisted depolymerization increases the selectivity of the reaction by breaking the lignin's compact bound with the help of a catalyst. Using microwave radiation and a ferric sulfate catalyst, Zhu et al. (2017) selectively depolymerized lignin into phenolic monomers, with phenolic dimers cleaving at a faster rate than non-phenolic dimers. Similarly, Anuar et al. (2021) used microwave heating to produce aromatic monomer (vanillin) and concluded that microwave heating is quick, extremely effective, homogeneous, selective, and environmentally friendly.

For lignin depolymerization, several recent types of research have used homogeneous catalytic systems, and while excellent results have been obtained for lignin model systems, many systems, meanwhile, are limited by the lack of selectivity (Behling et al. 2016; Schutyser et al. 2018) breakdown of the catalyst (Lange et al. 2013), as well as the need for harsh reaction conditions (Li et al. 2015). Furthermore, problems associated with the recycling of homogenous catalysts make their limited use on an industrial scale. Research has proven that heterogeneous catalysts are successful in attaining medium to high yields in the catalyzed oxidation of lignin compounds and monoaromatic chemicals, making them well-suited for commercial applications (Deng et al. 2010; Mottweiler et al. 2015; Sturgeon et al. 2014; Zhang et al. 2009).

Due to the high oxygen storage/release capacities, CeO_2_ has been widely used as a catalyst or catalytic support for various redox processes (Ahniyaz et al. 2004). According to Anuar et al. (2021), the high base characteristics of Ce/MgO catalysts improved the cleavage of *β-o*-4 in the lignin structure, enhancing the reaction's selectivity.

It has been widely reported that adding Zr, La, or W with CeO_2_ can increase its ability to store oxygen and its reducibility (Basile et al. 2019; Zhang et al. 2020). ZrO_2_ has an amphoteric nature because it possesses both acidic and basic properties. It is more chemically inert and more stable support under reducing conditions (Jaenicke et al. 2008). Ce–Zr mixed oxides have received extensive research as potential catalysts for a variety of lignocellulosic biomass transformations, such as lignin model compounds (Schimming et al. 2015), lignin itself (Yoshikawa et al. 2014) and cellulose conversion (Dar et al. 2015; Grams et al. 2016). Smaller particle sizes were formed more frequently when Zr^4+^ was added to the ceria lattice. The process of vacancy formation and oxygen mobility in the ceria lattice are both accelerated by the addition of Zr, which also boosts the material's reducibility (Nahar and Dupont 2014).

In the current work, we describe the synthesis of CeO_2_–CA, ZrO_2_–CA, and CeZrO_2_–CA, using the citrate complexation method. The characterization and preparation of the catalysts will be examined to identify any possible linkages between their physio-chemical characteristics, synthesis process, and catalytic activity in the oxidative depolymerization procedure. To further increase the lignin depolymerization efficiency in terms of lignin conversion, product yield, and product distribution, the impacts of depolymerization settings such as reaction temperature, reaction duration, catalyst loading, and pH are further explored. In addition, the variation in product yield brought on by the kenaf stalk and extracted lignin under the same operating conditions was also investigated. Moreover, the newly designed catalyst will help us to meet the Global demand for vanillin.

## Materials and methods

### Preparation of catalysts

The metal cations were complexed with the citrate ion to produce a citrate complex using nitrate precursors (Fuentes and Baker 2009). The cerium oxide citrate complex (CeO_2_–CA), zirconium dinitrate oxide citrate complex (ZrO_2_–CA) and mixed metal oxide citrate complex (CeZrO_2_–CA) were synthesized. Zirconium dinitrate oxide (99.9%, Alfa Aesar) and cerium nitrate hexahydrate (99.99%, Aldrich) were used as precursors. Each nitrate was separately dissolved in distilled water before the solutions were combined. The citric acid was added to the cation solution after being diluted in distilled water.

All formulations had a metal oxide to a citric acid molar ratio of 1:2, as shown in Table 1. The solution was mixed at room temperature to make a homogeneous solution. To eliminate extra water and transform the solution into a translucent gel, the mixture was heated to 80 °C and kept there while stirring. The solution thickened as the temperature rose, causing foam to develop; eventually, the combination gelled, leaving no turbidity or precipitation in its wake. The viscosity of the solution increased as the temperature was held at 80 °C, and both water and NO_2_ were evaporated. The precursor underwent initial thermal disintegration in the air for 1 h at 200 °C. The resulting ash-like substance was calcined at 500 °C for 1 h in a muffle furnace at a 5 °C/min heating rate.

**Table 1:** Composition of the synthesized catalysts

Composition	Nitrate precursors (NO_3_)_3_.6H_2_O)	Citric acid (C_6_H_8_O_7_)
CeO_2_–CA	0.5 M cerium nitrate hexahydrate (Ce (NO_3_)_3_ ∙ 6H_2_O)	1 M
ZrO_2_–CA	0.5 M Zirconium dinitrate oxide ZrO (NO_3_)_2_ ∙ xH_2_O	1 M
CeZrO_2_–CA	0.5 M cerium nitrate hexahydrate (Ce (NO_3_)_3_.6H_2_O) + 0.5 M Zirconium dinitrate oxide ZrO (NO_3_)_2_ ∙ xH_2_O	2 M

### Catalyst characterization

Panalytical XPert3 Powder X-ray diffraction (XRD) was used with monochromatic Cu K*α* radiation to investigate calcined powders. In step-scanning mode, data were collected for the angular range (20° to 80°) in steps of 0.02° with a step-counting time of 10 s. The FTIR spectrometer (Perkin Elmer; model Frontier 01) was used to record the outcomes in their as synthesized and annealed states from 4000 to 400 cm1. SSA measurements were obtained using Brunauer Emmett and Teller (BET) analysis by nitrogen adsorption (ASAP 2020, Micromeritics). The equation *d* = 6/*ρσ* was used to calculate the mean particle diameter (*d*), where *ρ* gives the solid density, and *σ* defines the BET-specific area of the solid solutions, respectively (Ahniyaz et al. 2004). Using field emission scanning electron microscopy (FESEM, Zeiss Supra 55VP), the resultant product's morphology was examined. Transmission electron microscopy (TEM, Hitachi, Model: HT7830 TEM) and an energy dispersive X-ray spectroscopy (EDS) analysis was used to obtain the pictures and elemental analyses of the materials. A 3 mm copper grid coated with a holey carbon sheet and a sample powder dispersed using ultrasound in hexane was used to produce samples for TEM analysis. These samples were then allowed to dry overnight before use. Temperature program oxidation (TPO, Brand: Thermo, Model: TPDRO 1100) of the samples were carried out to investigate the oxidation states. Furthermore, the used catalyst undergoes SEM and XPS analysis to evaluate the structural changes before and after the depolymerization reaction.

### Extraction of lignin from kenaf stalk

The lignin was extracted from kenaf fiber using the Soxhlet extraction method (Anuar et al. 2021; Orsi et al. 2022). The Soxhlet extraction process was used to grind and get the extract out of the kenaf stalks. The product was treated with NaOH to dissolve the lignin and hemicellulose linkages. The mixture that resulted from this process was heated in a hydrothermal vessel, cooled, and filtered to remove the solid residue. H_3_PO_4_ was used to acidify the filtered black liquid, and NaOH was used to precipitate the lignin. The precipitated lignin was filtered, washed, and dried. The lignin yield extracted from the kenaf stalk in this study is 25.7% using the mentioned equation (Eq. 1):

1 $$\mathrm{\%age\,yield\,of\,lignin}=\frac{\mathrm{Weight\,of\,lignin }\left(\mathrm{g}\right)}{\mathrm{ Weight\,of\,dreid\,Kenaf\,stalks }\left({g}\right)}\,\times\,100$$

### Catalytic oxidation of kenaf lignin by newly synthesized catalyst

**Fig. 1:**
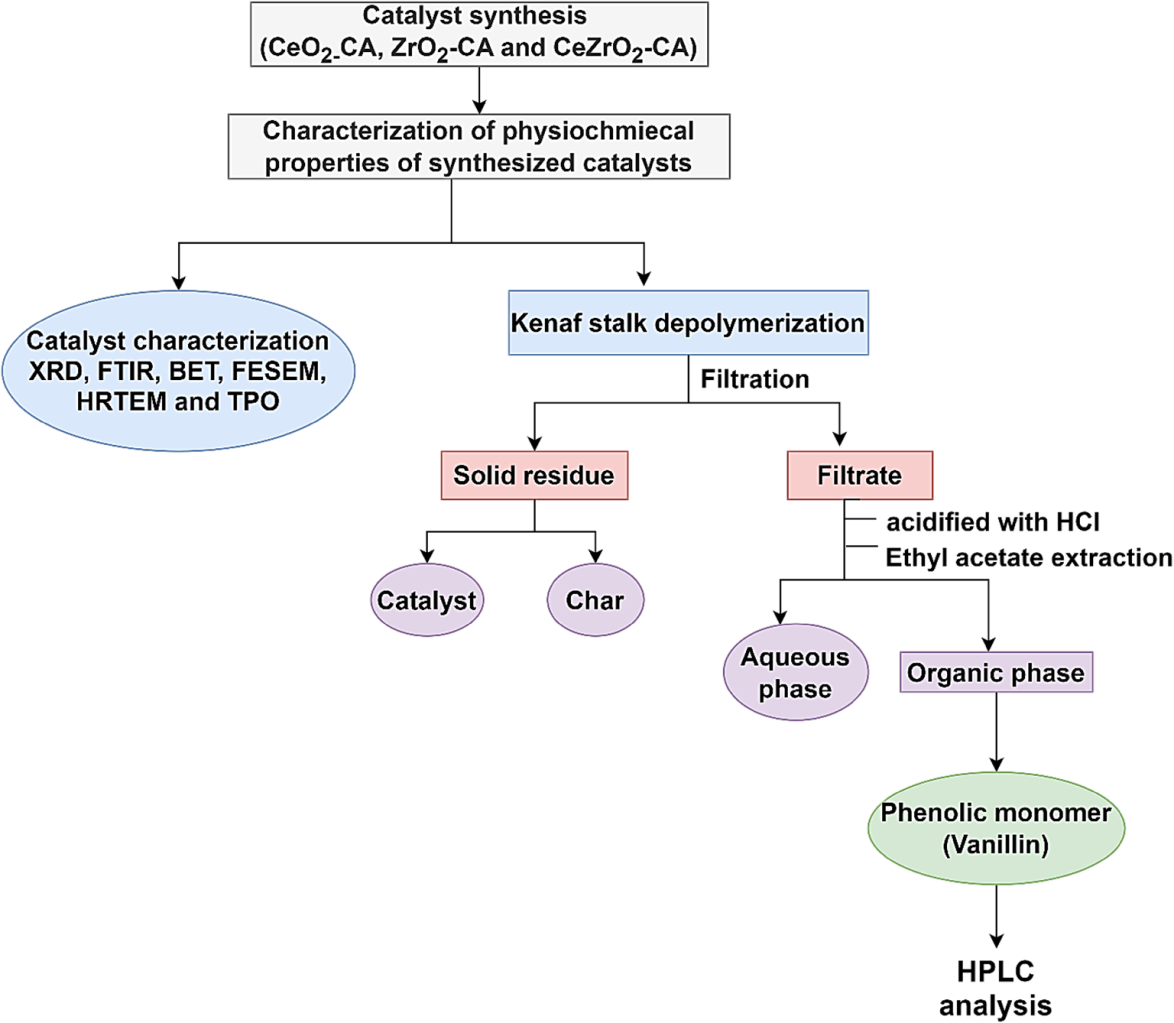
Experimental design schematic diagram

The dried kenaf stalk (1 g) and the extracted lignin (0.53 g) were soaked in a 0.01N NaOH solution (10 mL) overnight in an airtight container. A Teflon vessel was filled with 0.5 mL of H_2_O_2_, 0.05–0.15 g of catalyst (5–15 wt%), and a solution that had been soaked overnight. The mixture was vigorously stirred with a magnetic stirrer to produce a homogeneous solution. The Teflon container was heated at 160–180 °C for 10–30 min in a microwave reactor (Milestone Srl, Milan, Italy, MicroSYNTH MA143). The mixture was then allowed to cool to ambient temperature before being filtered using filter paper to remove the insoluble components. The residues were washed with 0.01N NaOH solution. The filtrate was placed into a sealed test tube. Concentrated hydroxide chloride (37%) acidified the filtrates, lowering the pH below 2.0. The mixture was then stirred for 15 s with a vortex agitator at 5000 rpm and then centrifuged for 15 min at 1000 rpm. The supernatant was then diluted with ethyl acetate (1:1). Vanillin was one of the low molecular weight molecules isolated into the organic phase. After being stirred for 60 s at 5000 rpm and then centrifuged for 5 min at 1000 rpm, the mixture split into two phases. The higher phase was put into a vial. A rotary evaporator extracted extra solvent at 50 °C and 400 mbar (Anuar et al. 2021; Qu et al. 2017)*.* Figure 1 illustrates an experimental design schematic diagram. To further test the reusability of the catalyst under optimal reaction conditions, the catalyst residue was washed many times with ethanol at the same time. This was followed by a drying procedure in an oven at 100 °C for 2 h.

### High-performance liquid chromatography (HPLC) analysis

Agilent high-performance liquid chromatography (HPLC) with a UV–Vis detector operating at a wavelength of 280 nm was used to evaluate the brownish solution containing vanillin. The Santa Clara, California, United States-based HPLC (Agilent 1200 series) was fitted with a Hypersil C18 column (size distribution: 5 m, 150 4.6 mm inner diameter). The mobile phase was acetonitrile: water (1:8 v/v) with 1% acetic acid at a flow rate of 2 mL min^−1^, and the column temperature was fixed at 35 °C. Vanillin (Sigma-Aldrich, purity 99.7%, Darmstadt, Germany) was employed as the standard.

### Gas chromatography–mass spectrometry (GC–MS) analysis

The precise composition of the product was examined using qualitative gas chromatography–mass spectrometry (Agilent 7890 A) on a DB-5MS column (30 m 0.25 mm i.d.; 0.25 m film thickness) at an ionization voltage of 70 eV. The oven was set to heat to 50 °C for 3 min, then to 300 °C at 8°C min^-1^ and hold that temperature for 20 min. The detector was set to 300 °C, whereas the injection component's temperature was 250 °C. The carrier gas was helium (He), which was employed at 1.6 mL min^-1^ with a 1 L injection volume. According to Qu et al. (2017), the split ratio was 1:10 and the mass range (m/z) ranged between 40 and 600 m/z.

## Results and discussion

### Catalyst characterization

**Fig. 2:**
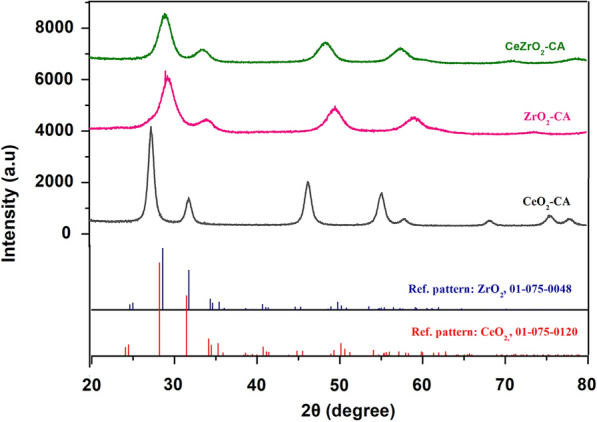
XRD pattern of synthesized catalysts

The XRD model of CeO_2_–CA, ZrO_2_–CA, and CeZrO_2_–CA produced using the citrate complexation approach is illustrated in Fig. 2 after being calcined at 500 °C for 1 h. The calcined CeO_2_–CA, ZrO_2_–CA and CeZrO_2_–CA nanocomposites are indexed using reference pattern: Cerianite, 98-007-2155 for cerium oxide (crystal framework: cubic, *a*/*b*/*c* = 5.412 A° and *α*/*β*/*γ* = 90°) and reference pattern: Baddeleyite, 01-079-1796 for zirconium oxide (crystal framework: Orthorhombic; *a* = 5.068A°, *b* = 5.2600A°, *c* = 5.077A° and *α*/*β*/*γ* = 90°). The cubic phases of CeO_2_ with fluorite structure were detected by the diffraction peaks at 28.74°, 47°, and 56.2° (ref. JCPDS card 98-007-2155) (Ghosh et al. 2020). At 34°, 50.3°, and 60.1°, respectively, the distinctive peaks of ZrO_2_'s orthorhombic phases were found (ref. JCPDS card 01-079-1796)(Yaacob et al. 2019).

Despite this, the mixed oxide sample's lattice parameter differs from CeO_2_'s bulk oxide parameter. Lattice parameters for CeZrO_2_–CA sample are *a* = 5.27A° and *c* = 5.29A°, suggesting alteration and flaws in the crystallite structure that can be attributed to oxygen vacancies since the enthalpy of the creation of oxygen vacancies reduces with the shrinkage of crystallite sizes. Due to the loss of one oxygen atom, two Ce^4+^ atoms must be replaced by Ce^3+^ atoms to maintain the lattice's neutrality. However, because the Ce^3+^ ionic radius (0.114 nm) is larger than the Ce^4+^ ionic radius (0.097 nm), the distances between the bonding atoms must be modified, which changes the lattice characteristics.

The broadening of (111) and less intense peaks are shown by the processing of the ceria–zirconia samples. The primary peak's breadth reveals the size of the crystallites, both large and small. The wide peaks are linked to enormous crystallite size, and vice versa (Hadi et al. 2015; Jani et al. 2013). In addition, the mixed oxide composite's XRD pattern shows that CeO_2_ and ZrO_2_ coexisted in their distinctive peak locations, indicating that both ZrO_2_ and CeO_2_ were present in the composite (homogenous composite). The XRD lines of the mixed oxides are comparable to CeO_2_ and ZrO_2_, showing that zirconia substitution has stabilized the fluorite structure. The XRD pattern of calcined samples becomes more concentrated with cerium oxide and zirconium oxide formation. Furthermore, the XRD results show no undesirable peaks, which excludes the presence of contaminants in the processed samples. Numerous research also reported on the impact of the metal oxide calcination process on the growth of crystal size and improved cluster building (Chen et al. 2010; Gaber et al. 2014).

The Debye–Sherrer method (*S* = 0.9*λ*/*β* cos *θ*) was employed to determine the crystallite size (*S*, nm) of CeO_2_–CA, ZrO_2_–CA and CeZrO_2_–CA after being calcined at 500 °C, (Ali et al. 2021a; Holzwarth and Gibson 2011). The crystalline size of nanocomposites was determined to be 1.63 nm, 0.60 nm, and 0.874 nm for CeO_2_–CA, ZrO_2_–CA, and CeZrO_2_–CA, respectively (Table 2). It clarifies how the small crystalline size of nanocomposites has a significant surface area which ultimately helps to break down lignin bonds to produce aromatic compounds.

**Table 2:** Catalyst crystallite size after a 1-h calcination at 500 °C

Catalyst	Angle 2*θ* main peak (111)	Lattice parameters (A°)		Crystalline size (nm)
		*a*	*c*	
ZrO_2_–CA	29.33	5.06	5.07	0.60
CeO_2_–CA	28.89	5.412	5.412	1.63
CeZrO_2_–CA	29.01	5.27	5.29	0.874

**Fig. 3:**
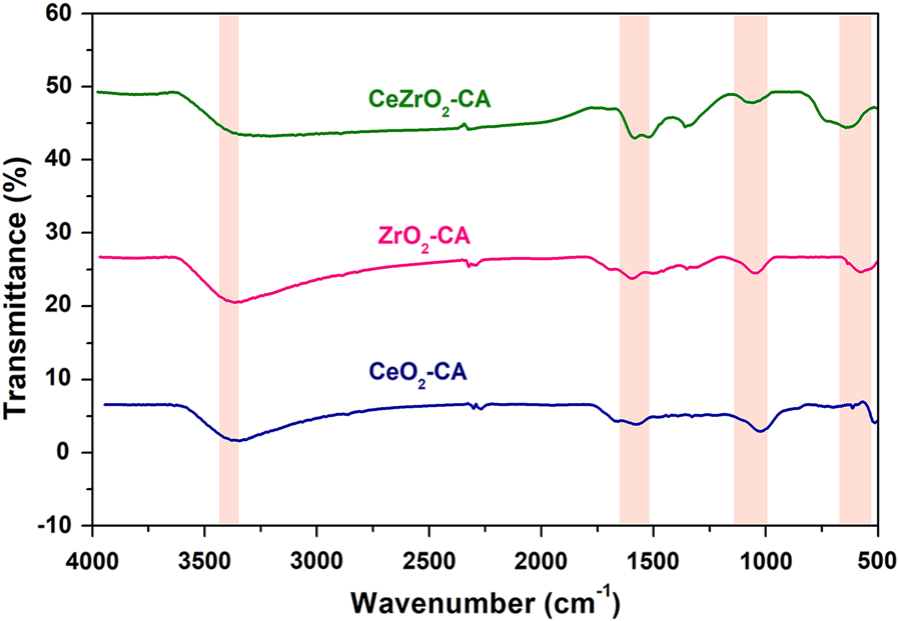
FTIR analysis of catalysts

After calcination at 500 °C, the FTIR spectra of nanocomposites made of CeO_2_–CA, ZrO_2_–CA, and CeZrO_2_–CA are shown in Fig. 3. The resultant nanocomposites have moderate absorption bands at 3400–3425 cm^−1^, 1625–1680 cm^−1^, 1020–1140 cm^−1^, and 1520–1550 cm^−1^ that are synchronized to the stretching and bending vibrations of hydroxyl groups, Ce–O–Ce, C–O, and C–H aliphatic, respectively (Ali et al. 2021b). The stretching and bending vibration modes of Zr–O and Ce–O within the nanocomposites correlate to the strong absorption bands in the 400–800 cm^−1^ range (Ali et al. 2021a; Pouretedal et al. 2012). The stable CeO_2_–CA, ZrO_2_–CA, and CeZrO_2_–CA nanocomposites are produced because of the data extracted from FTIR spectra (500–4000 cm^–1^). The bonding of the hydroxide groups on the surface of the nanocomposites provides a reasonable explanation for the surface activity of the produced nanocomposite.

**Fig. 4:**
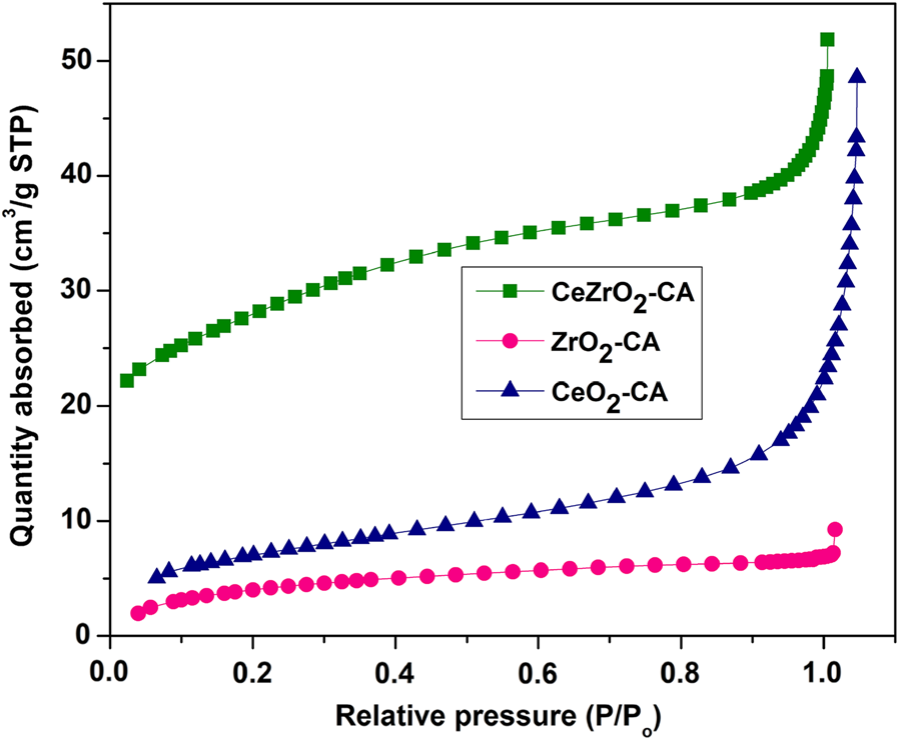
Adsorption isotherms of catalysts

The synthesized catalysts' nitrogen gas adsorption isotherm distribution is shown in Fig. 4, which represents the type IV (mesoporous) isotherm according to the IUPAC classifications. The marginally increased absorption of N_2_ at P/P_o_ in the range of 0.1–0.9 indicated it also possessed mesopores, and higher uptakes of N_2_ existed in a tiny quantity of macropores. This finding showed that the calcination ceria–zirconia sample had a higher likelihood of exhibiting mesopores due to the phenomena of micropore collapse throughout the calcination process, which led to an increase in pore size. The layouts of the isotherms and hysteresis loops were used to identify the porous features in the manufactured samples. The presence of a pore outlet or the size of the pores is what causes the hysteresis to emerge from the isotherm profile. As a result, the samples' isotherms had a sizable amount of hysteresis, which was explained by the existence of mesoporous structures.

Table 3 summarizes all synthesized catalysts' surface area, pore volume, and pore size. The CeZrO_2_–CA catalyst shows a higher surface area than the other synthesized catalyst. Zirconia doping of ceria decreases the temperature of the surface and aggregate reduction by increasing the number of oxygen vacancies (Ali et al. 2021a; Schimming et al. 2015). While compared to CeO_2_–CA, ZrO_2_–CA and CeZrO_2_–CA catalysts had reduced average pore sizes and pore volumes. It also demonstrates that the high-temperature calcination process decreased the specific surface area and increased pore size. These findings support the already published research on the connection between calcination and the size of pores (Hadi and Yaacob 2007; Jani et al. 2013). The synthesis of catalyst with its promising properties of greater surface area, excellent adsorption, and narrow pore size distribution results in an increase in catalytic activity.

**Table 3:** Textural properties of ZrO_2_–CA, CeO_2_–CA, and CeZrO_2_–CA

Catalyst	Surface area (m^2^/g)	Pore volume (cm^3^/g)	Pore size (nm)
ZrO_2_–CA	26	0.016	2.557
CeO_2_–CA	28	0.06	4.94
CeZrO_2_–CA	50	0.05	10.52

**Fig. 5:**
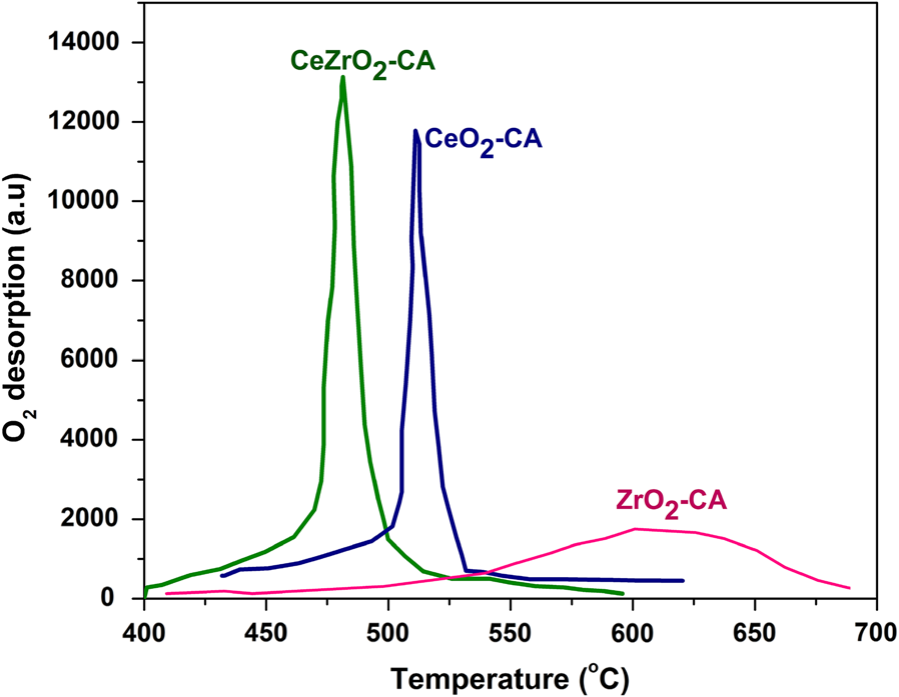
TPO curves of catalysts

In contrast, O_2_-TPO studies were carried out to demonstrate the oxygen intake and storage capability in relation to temperature, as shown in Fig. 5 (Bueno-López et al. 2005). Since CeO_2_ is known for its ability to store oxygen so it is believed that the O_2_ reaction involves the lattice oxygen of CeO_2_–CA at the temperature range of 400–700 °C (Ramli 2023). The results clearly show that the intensity of oxygen intake increased in the following order: CeO_2_–CA < ZrO_2_–CA < CeZrO_2_–CA. The CeZrO_2_–CA has a higher specific surface area (50 m^2^/g), which ensures good interaction between the oxygen particles and the catalyst (Devaiah et al. 2018). The surface areas of CeO_2_–CA and ZrO_2_–CA are 28 m^2^/g and 26 m^2^/g, respectively, showing less interaction. CeZrO_2_–CA has the greatest number of poorly bound oxygenated species because of its highly oxidized surface, according to research published by Torrente-Murciano et al. 2016.

**Fig. 6:**
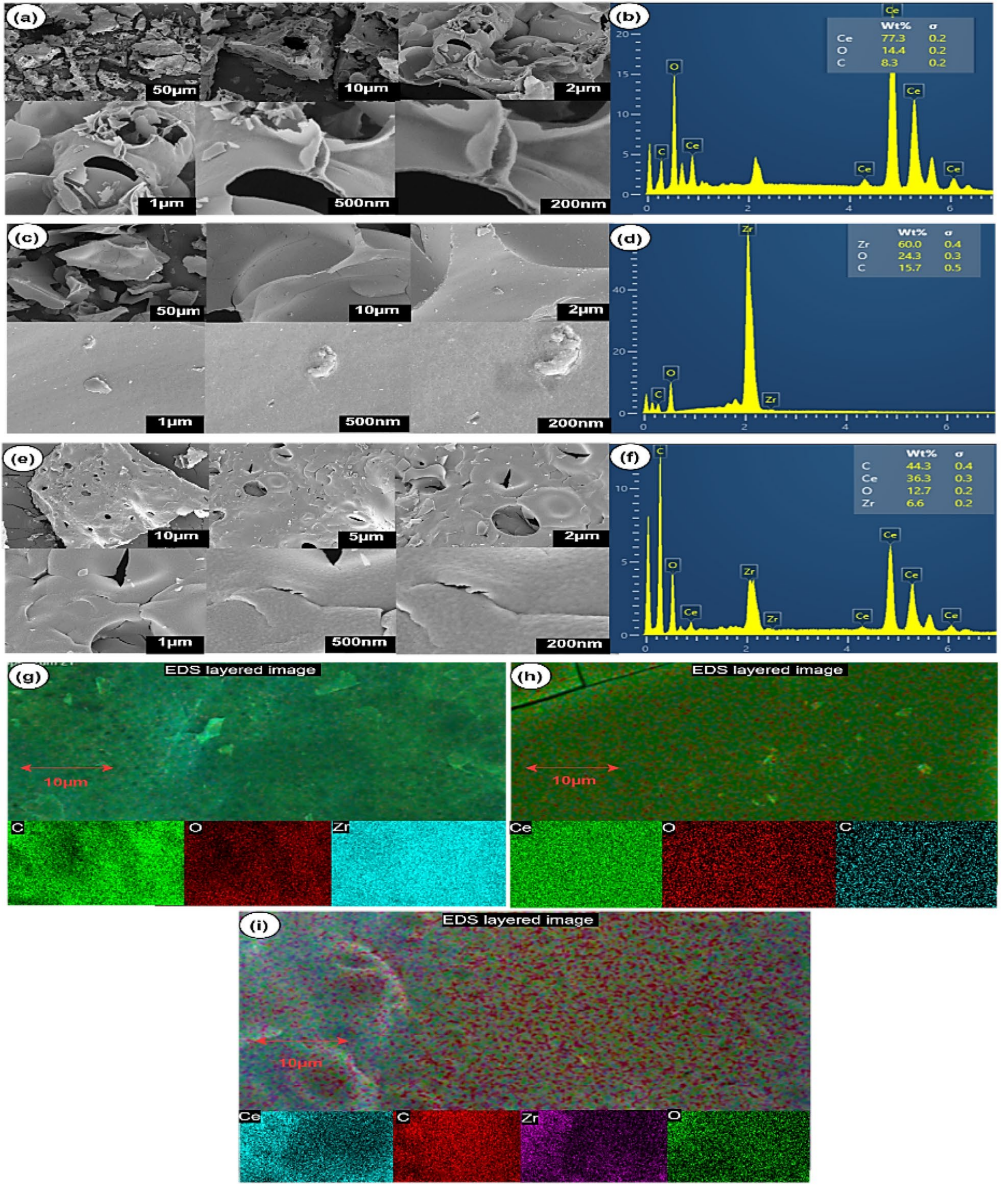
FE–SEM images of the calcined nanocomposite: CeO_2_–CA (**a**), ZrO_2_–CA (**c**), and CeZrO_2_–CA (**e**) samples, EDX pattern of CeO_2_–CA (**b**), ZrO_2_–CA (**d**), and CeZrO_2_–CA (**f**) samples and the EDXS mapping of CeO_2_–CA (**g**), ZrO_2_–CA (**h**), and CeZrO_2_–CA (**i**)

**Fig. 7:**
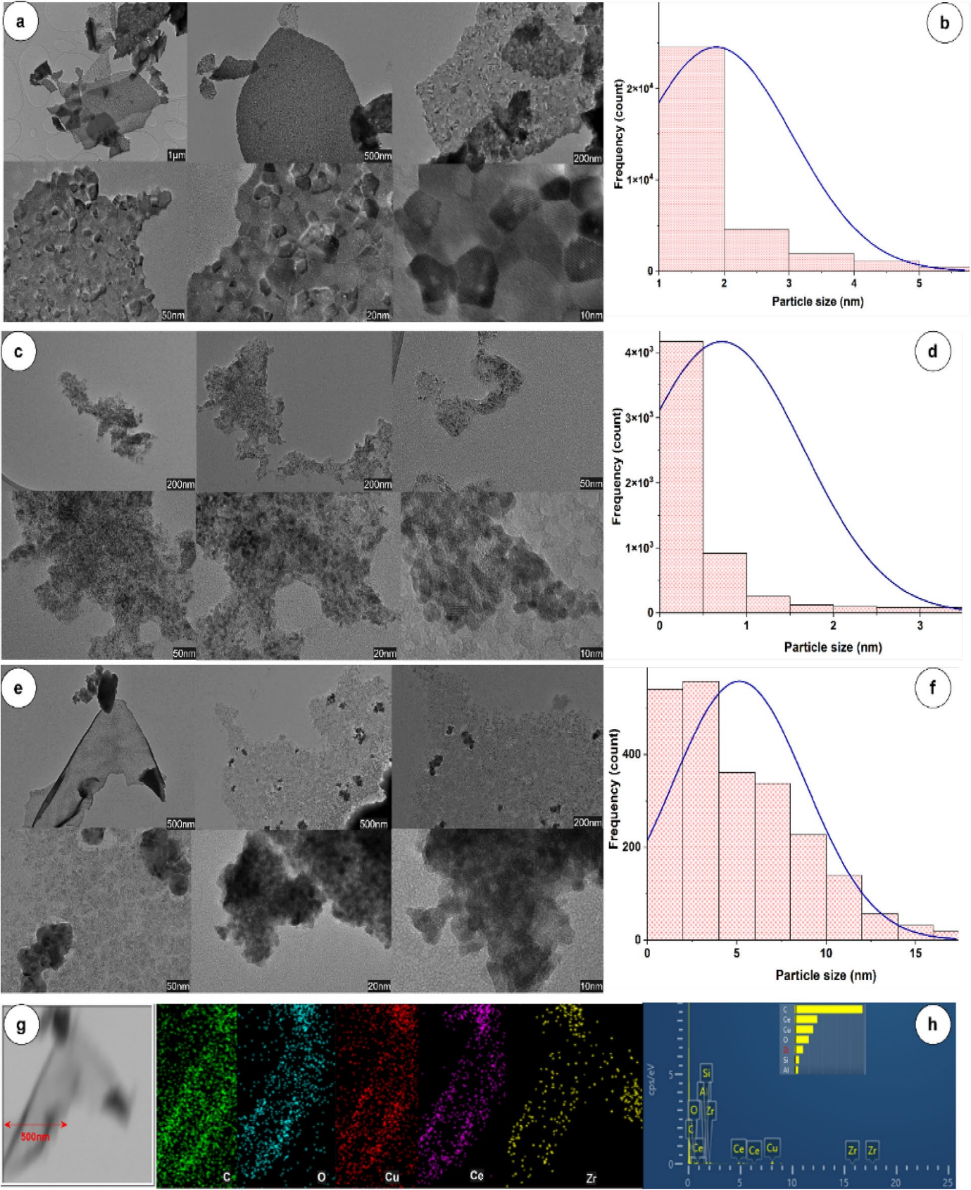
HR–TEM images of the calcined nanocomposite: CeO_2_–CA (**a**), ZrO_2_–CA (**c**), and CeZrO_2_–CA (**e**) samples, histograms of CeO_2_–CA (**b**), ZrO_2_–CA (**d**), and CeZrO_2_–CA (**f**) samples and the EDXS mapping and pattern of CeZrO_2_–CA (**g**, **h**), respectively

The morphology studies of CeO_2_–CA, ZrO_2_–CA and CeZrO_2_–CA are examined through FESEM and TEM, shown in Figs. 6 and 7, respectively. Figure 6 presents FESEM pictures of a calcined powder at 500 °C. These images demonstrate the characteristic shape of Ce, Zr, and Ce–Zr mixed oxides synthesized through the citrate complexation process. The morphologies of CeO_2_–CA (Fig. 6a) and ZrO_2_–CA (Fig. 6c) are very different from one another. Compared to CeO_2_–CA (Fig. 6a), which has spherical aggregates (Ali et al. 2021b), ZrO_2_–CA (Fig. 6c) are distinguished by their angular shape (Fuentes and Baker 2009). There is a significant amount of agglomeration in both situations. The morphology of CeZrO_2_–CA (Fig. 6e) is spongy, having pores ranging from 12 to 13 nm in diameter. All three samples are made up of bubbles or holes with walls of materials separating them, as can be seen in the images at increased magnification.

CeO_2_–CA, ZrO_2_–CA and CeZrO_2_–CA samples' elemental analyses were examined using the EDX approach, as shown in Fig. 6b, d, e, respectively. The four lines were illustrated due to the presence of elements, such as Zr, Ce, C, and O. Furthermore, it shows that pure CeO_2_–CA (Fig. 6b), ZrO_2_–CA (Fig. 6d) and CeZrO_2_–CA (Fig. 6f) nanocomposites were successfully synthesized. Carbon was visible due to using the carbon grid during the FESEM/EDX technique. The EDXS mapping of the samples is shown in Fig. 6g, h, i. The distribution of the cerium, zirconium, oxygen, and carbon (due to grid) components in the synthesized nanocomposite appears satisfactory. In the CeZrO_2_–CA sample (Fig. 6i), the zirconium oxide was uniformly distributed inside the cerium oxide, according to EDS layered mapping. In addition, it agreed with the data gathered from the EDX pattern of the CeZrO_2_–CA sample (Fig. 6f), which indicated that the nanocomposite contained a mixture of zirconium and cerium oxides.

Figure 7 shows the results of HR–TEM analysis for CeO_2_–CA, ZrO_2_–CA, and CeZrO_2_–CA nanocomposites. According to Fig. 7, the sample's CeO_2_–CA and CeZrO_2_–CA HR–TEM images revealed hard, dense agglomerations with regular and uneven spherical forms (a, e). Figure 7 displays the data that was gathered and used to generate the histogram graph for CeO_2_–CA, ZrO_2_–CA, and CeZrO_2_–CA [(b), (d), (f)], respectively. The average particle size of the CeO_2_–CA sample is calculated to be 1.88 nm based on the TEM picture and histogram graph (Fig. 7a, b). While the average particle size of the sample ZrO_2_–CA is assessed to be 0.71 nm based on the TEM picture and histogram graph (Fig. 7c, d). The ZrO_2_–CA sample's predicted particle size differed from the measured crystallite size. The produced cerium oxide (CeO_2_–CA) and cerium–zirconium oxide composite (CeZrO_2_–CA) particles manifested as dense agglomerations. According to HR–TEM images (Fig. 7e), the calcined CeZrO_2_–CA sample was composed of a soft collection of spherical particles with an average particle size of about 5.14 nm. In addition, the collected data were used to create the histogram graph shown in Fig. 7f.

The CeZrO_2_–CA sample's estimated particle size diverged from its anticipated crystallite size due to dense agglomerations of the composite's cerium oxide and zirconium oxide particles (the CeZrO_2_–CA sample) developed.

### Evaluation of catalytic performance

**Fig. 8:**
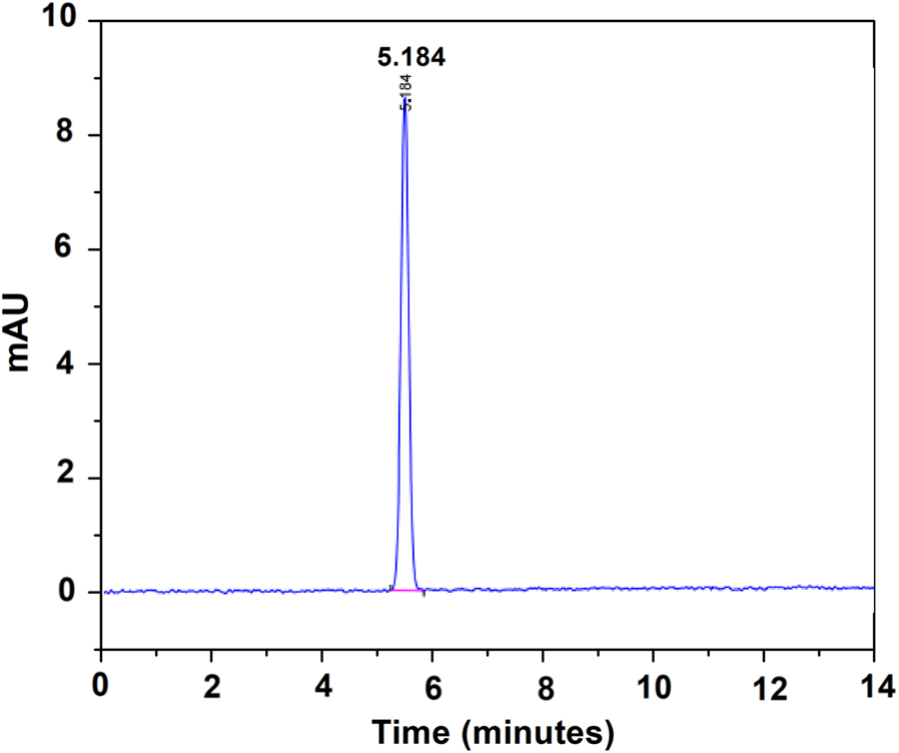
HPLC chromatogram of vanillin standard

By considering the retention time of derived vanillin to the standard vanillin, vanillin yield was determined and characterized using high-performance liquid chromatography (HPLC). The HPLC chromatogram of the vanillin standard is shown in Fig. 8, revealing that the vanillin retention time is 5.184 min.

**Fig. 9:**
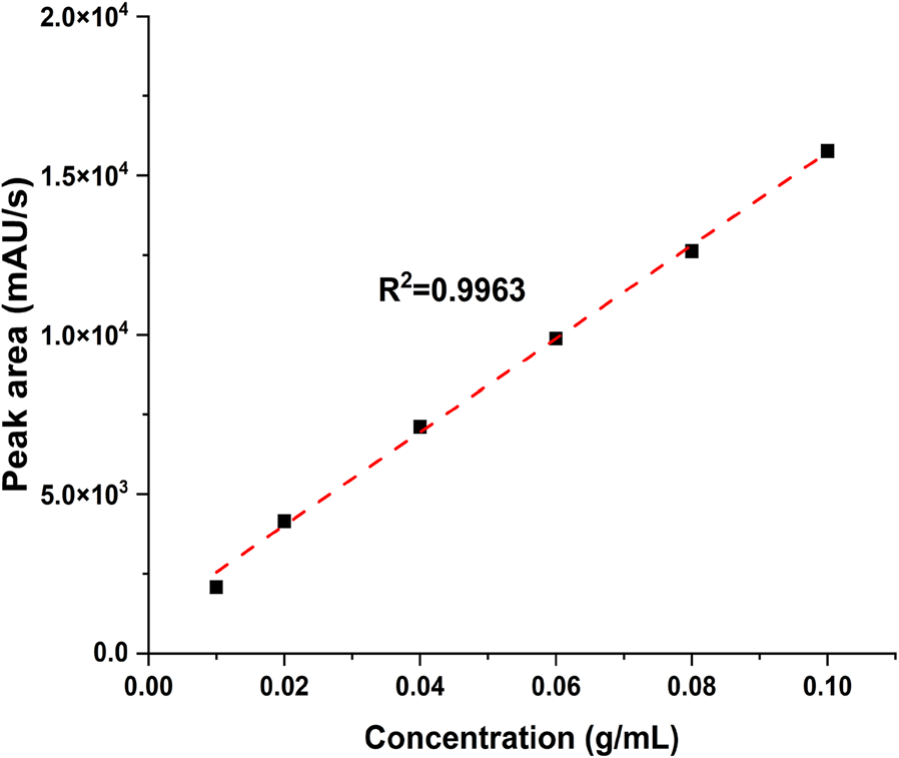
Calibration curve of the vanillin standard at five different concentrations

This chromatogram served as the basis for preparing five different quantities of standard vanillin for the calibration curve, enabling the quantitative measurement of the vanillin generated through the catalytic depolymerization of the kenaf stalk. Figure 9 displays the calibration curve for the vanillin standard via HPLC at five different concentrations (1.25 ⨯ 10^–4^, 6.25 ⨯ 10^–5^, 3.125 ⨯ 10^–5^, 1.56 ⨯ 10^–5^ and 7.81 ⨯ 10^–6^). The peak area from the injected aliquot of standard vanillin solution with known concentration was measured by plotting the curve. The vanillin yield will be quantified using the calibrated curve. The best-fit line's correlation coefficient, or R^2^, indicates high accuracy, which is at 0.9963.

**Fig. 10:**
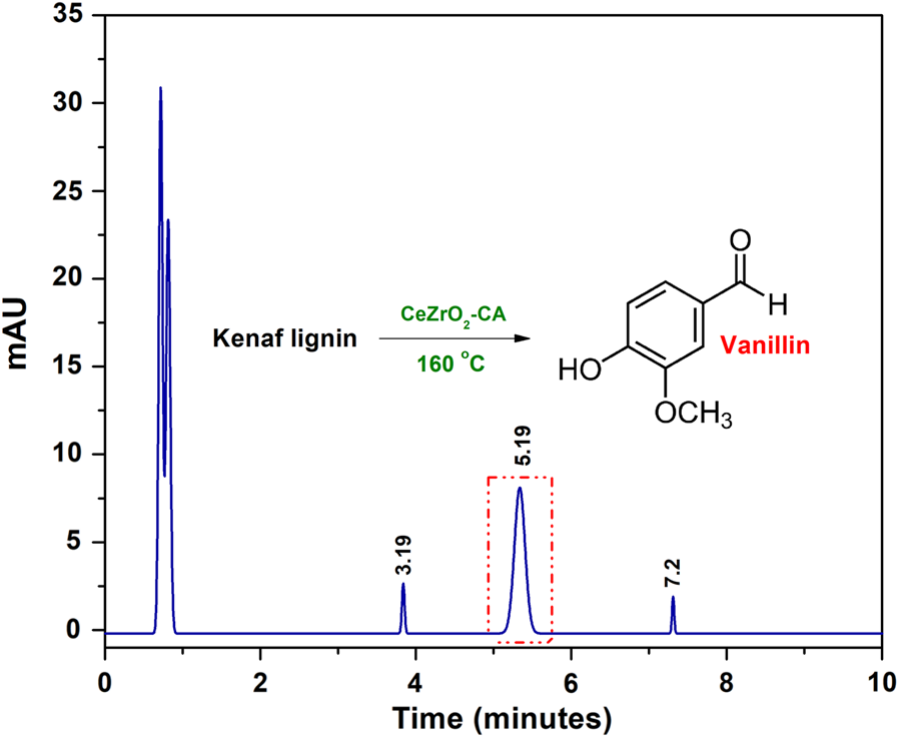
HPLC chromatogram of derived vanillin

Vanillin was found with a retention time of 5.18 min in the liquid utilising CeZrO_2_–CA as a catalyst when employed to oxidatively depolymerize the kenaf stalk (Fig. 10). Three more compounds, with retention times of 0.7, 3.9, and 7.1 min, are also visible on the chromatogram. The other substances might be 4-vinyl guaiacol, syringol, and syringaldehyde. Table 4 presents the results of lignin depolymerization into vanillin under consistent conditions: 5% catalyst loading, 160 °C temperature, and 30 min of microwave treatment.

**Table 4:** Evaluation of catalytic performance for vanillin production

Catalyst	Vanillin yield (%)
Without catalyst	Not detected
CeO_2_–CA	9.2%
ZrO_2_–CA	9.4%
CeZrO_2_–CA	9.9%

Based on the evaluation findings, it can be verified that vanillin was found in every synthetic catalyst's liquid output. The maximum vanillin yield (9.9%) was obtained using CeZrO_2_–CA as a catalyst at 160 °C temperature and 30 min of reaction time. The results suggest that CeOZrO_2_–CA contributed to accelerating the oxidative depolymerization of the kenaf stalk. The capacity of Ce to selectively cleave the C–C bonds in the kenaf stalk may be responsible for the more excellent lignin conversion and boosted vanillin production. In addition, prior studies have used a Ce/MgO catalyst to directly oxidize kenaf stalks (Anuar et al. 2021), producing a high yield of vanillin (3.70%). Ce is a successful catalyst with the potential to break the chemical bonds in the lignin structure.

Compared with low surface area catalysts such as CeO_2_–CA and ZrO_2_–CA, high surface area catalysts CeZrO_2_–CA accelerated the reaction according to factors impacting the reaction rate. The effects of vanillin synthesis on many factors, including reaction temperature, reaction time, catalyst loading, lignin extraction, and reaction pH, were studied through optimization studies. To evaluate the effectiveness of the catalyst, the biomass mass (2 g), biomass-to-solvent ratio (1:10 g/mL), and peroxide volume (1 mL) were all fixed.

### Effect of reaction temperature on vanillin production

**Fig. 11:**
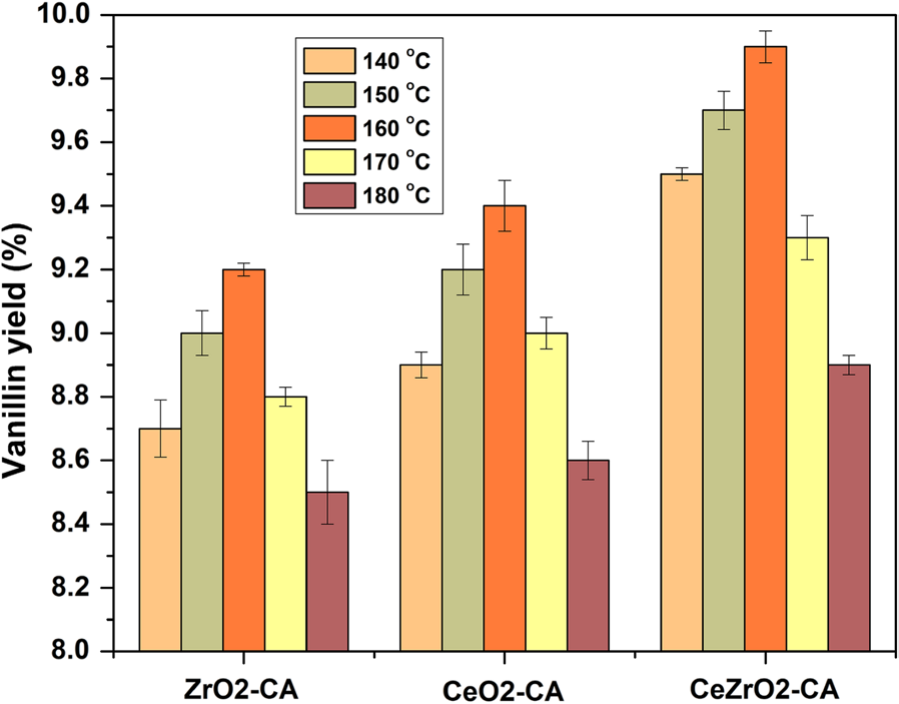
Effect of reaction temperature on vanillin production

The effect of reaction temperature on the depolymerization of kenaf stalk to produce vanillin was analyzed in a liquid using CeO_2_–CA, ZrO_2_–CA, and CeZrO_2_–CA as a catalyst with a constant reaction time of 30 min. The outcomes are displayed in Fig. 11. The findings demonstrate that temperature significantly influenced the distribution of vanillin yield. Based on the data, it was found that using CeZrO_2_–CA, the yield of vanillin increased from 9.5 wt% to 9.9 wt% when the reaction temperature was increased from 140 to 160 °C. However, the yield dramatically decreased from 9.9 wt% to 8.9 wt% when the temperature was further increased from 160 to 180 °C. A similar pattern was observed with CeO_2_–CA and ZrO_2_–CA, where the yield of vanillin increased from 8.9 wt% to 9.4 wt% at a temperature range of 140–160 °C and decreased from 9.4 wt% to 8.6 wt% at a temperature range of 160–180 °C for CeO_2_–CA. Similarly, for ZrO_2_–CA, the yield increased from 8.7 wt% to 9.2 wt% at temperature range of 140–160 °C and decreased from 9.2 wt% to 8.5 wt% at a temperature range of 160–180 °C. These findings suggest that the depolymerization of the kenaf stalk to produce vanillin is an exothermic process. Temperature management is crucial for the safe and effective conversion of biomass to vanillin (Aarabi et al. 2017). However, independent of the catalyst used, the vanillin yield dropped when the reaction temperature was raised to 180 °C. Higher temperatures may encourage side reactions, evaporation, and degradation and move the equilibrium back toward the source material, reducing the vanillin yield, as explained by (Anuar et al. 2021; Ito et al. 2020).

### Effect of reaction time on vanillin production

**Fig. 12:**
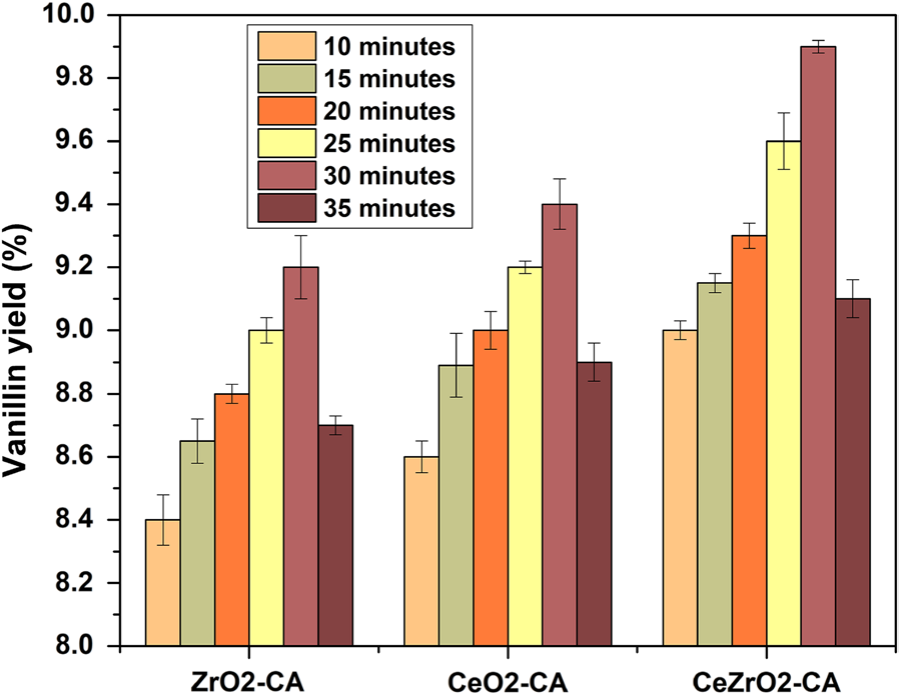
Effect of reaction duration on vanillin production

The effect of reaction duration on vanillin production is depicted in Fig. 12. The reaction was carried out using CeO_2_–CA, ZrO_2_–CA, and CeZrO_2_–CA as the catalyst with different reaction times (e.g., 10, 15, 20, 25, 30, and 35 min) at identical reaction temperatures (160 °C) which showed the highest yield in Fig. 11. The vanillin yield is increased gradually for all the catalysts when time increases from 10 to 30 min. When the reaction was carried out for 30 min at 160 °C, the CeZrO_2_–CA catalyst produced the maximum amount of vanillin (9.9%), while ZrO_2_–CA and CeO_2_–CA produced vanillin (9.1 wt% and 9.4 wt%, respectively). Bond cleavage had to take place to produce vanillin, which required enough heat without deteriorating the vanillin produced at the final phase of the oxidation cycle. Increasing the reaction time to 30 min leads to a significant decrease in vanillin yield. This could be due to over-oxidation. Longer reaction times can result in higher conversion but lower selectivity, according to Zhu et al., (2017), who explain simultaneous reactions. This leads us to the conclusion that the ideal reaction time for vanillin to reach its peak is 30 min.

### Effect of catalyst loading on vanillin production

**Fig. 13:**
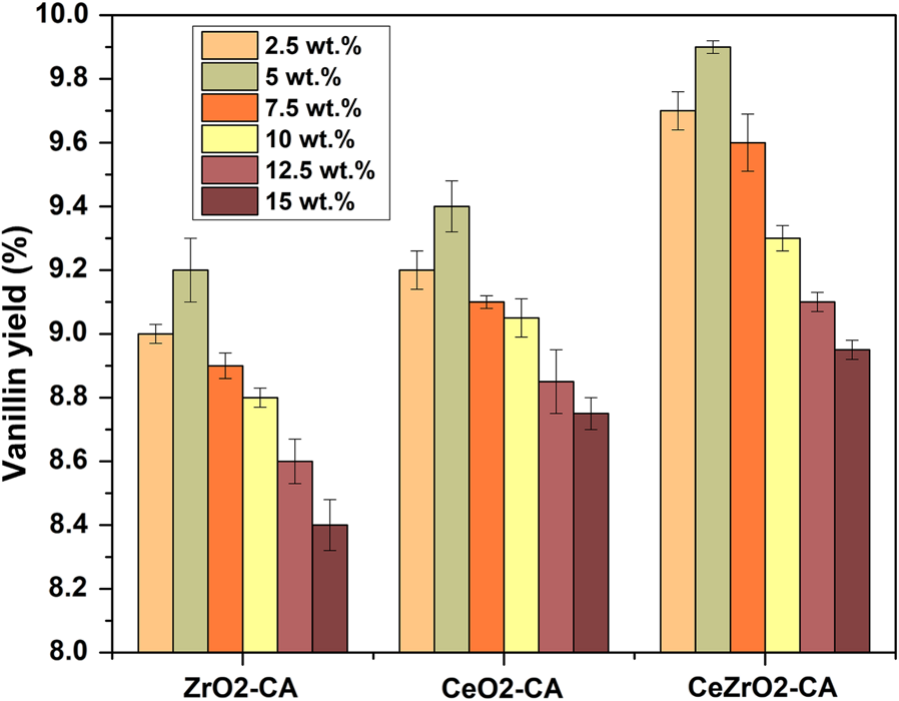
Effect of catalyst loading on vanillin production

The yield of Vanillin produced using various amount (2.5 wt%, 5 wt%, 7.5 wt%, 10 wt%, 12.5 wt%, and 15 wt%) of CeO_2_–CA, ZrO_2_–CA and CeZrO_2_–CA catalyst loading is shown in Fig. 13. The catalyst was synthesized using 2 g of dried Kenaf stalks and heated in a microwave for 30 min at 160 °C. The results showed that increasing the catalyst loading led to lower yields of vanillin, with yields of 9.7% (2.5 wt%), 9.9% (5 wt%), 9.6% (7.5 wt%), 9.3% (10 wt%), 9.1% (12.5 wt%) and 8.8% (15 wt%) using CeZrO_2_–CA catalyst. The vanillin yield drops when the catalyst loading is increased to 5 wt%. This could be because vanillin was overoxidized after fully forming with the oxidizing species in the media. The highest vanillin yield is recorded with 5 wt% of catalyst loading. Comparatively, to other supported catalysts, CeZrO_2_–CA had a large surface area and offered many active surfaces for the oxidation process of lignin.

### Analysis of vanillin production using CeZrO_2_–CA with extracted lignin

This work observed a high vanillin yield of 9.9% for 5 wt% of CeZrO_2_–CA at 160 °C for 30 min. As a result, 5 wt% of CeZrO_2_–CA was selected as the ideal catalyst for the direct oxidative depolymerization of vanillin from Kenaf stalks as it shows the higher yield (Fig. 13). Moreover, due to its ability to store oxygen, high mobility, redox stability, and adaptable surface characteristics, CeZrO_2_–CA is employed to explore further the impact of pH, catalyst reusability and vanillin production from extracted lignin.

### Effect on vanillin production with extracted lignin or without extracted lignin (kenaf stalk)

**Fig. 14:**
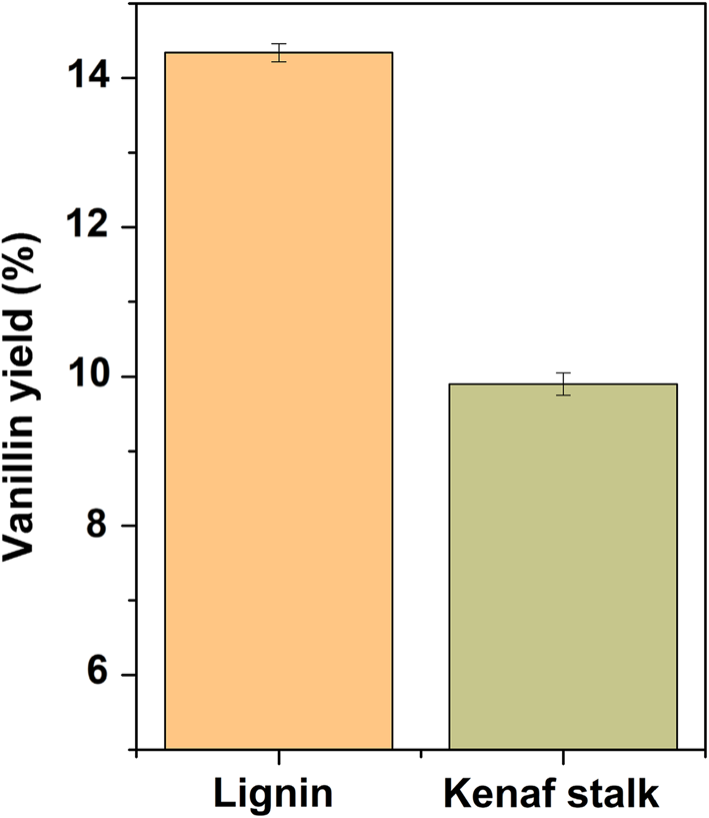
Vanillin production with extracted lignin or without extracted lignin (kenaf stalk)

According to Fig. 14, vanillin can be produced from biomass and extracted lignin by utilizing a 5 wt% of CeZrO_2_–CA as a heterogeneous catalyst for 30 min at 160 °C. In the presence of CeZrO_2_–CA catalysts, the yields of vanillin for the direct oxidation of biomass and the oxidation of extracted lignin were 9.9% and 14.3%, respectively. Because lignin was isolated as an active ingredient for converting biomass into vanillin, extracted lignin produced a higher yield than direct biomass. Due to the oxidation process, aryl ether linkages between *β*-O-4 bonds and C–C cleavage between lignin structures are broken down, which results in the generation of Vanillin. Since the lignin structure is not isolated from cellulose as well as hemicellulose components, it is difficult to cleave (*β*-O-4) bonds in direct biomass oxidation, which results in lower vanillin yield compared to the extracted lignin (Jiang et al. 2019; Lu et al. 2016).

### Effect of pH on vanillin production

Considerable alkalinity is required to selectively oxidize lignin's into aromatic aldehydes (vanillin) (Evgenievich et al. 2016). However, vanillin's stability can be influenced by pH; higher pH values cause faster breakdown and a lower vanillin yield. To increase vanillin productivity and quality, it is crucial to maintain an ideal pH (Tarabanko and Tarabanko 2017). Increasing the yield of aromatic aldehydes is the primary goal of using catalysts to oxidize lignin's.

**Fig. 15:**
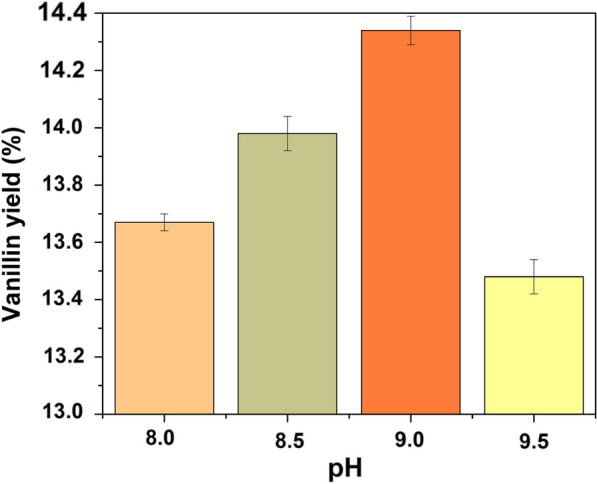
Effect of pH on vanillin production

Figure 15 displays the vanillin production obtained following oxidative depolymerization of the kenaf stalk at various pH levels. Utilizing NaOH, an alkaline media for vanillin was created. The pH ranges used were 8.0, 8.5, 9.0, 9.5, while the other reaction parameters were retained at 5 wt% of CeZrO_2_–CA, 160 °C, and 30 min, respectively. The vanillin production dramatically increased from 9.1% to 9.9% as the pH moved from 8.0 to 9.0, but it significantly dropped to 9.4% when the pH moved from 9.0 to 9.5. The reaction was also carried out at pH 9.0, which itself is lower than the pH range of 11–14 documented by other studies (Anuar et al. 2021; Araújo et al. 2010; Fache et al. 2016).

### Recyclability of catalyst on vanillin production

**Fig. 16:**
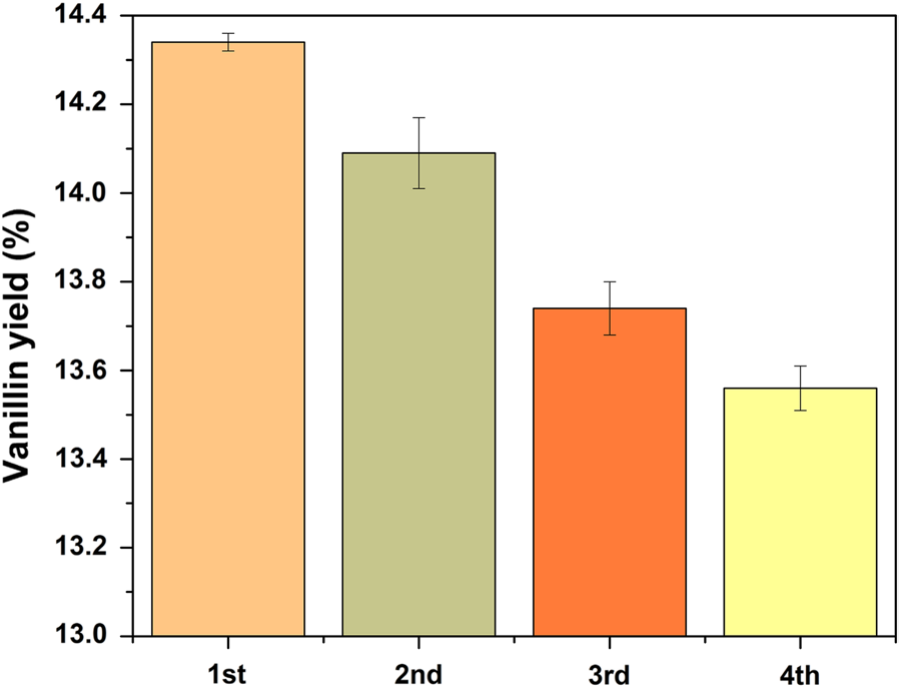
Recyclability of catalyst on vanillin production

A crucial factor in determining the performance of catalysts is their stability. Therefore, in the present research, the strength of the catalysts was examined in terms of their ability to be recycled. Following the vanillin production step, the utilized catalyst was filtered away from the unreacted biomass before being washed with ethanol and water. The isolated catalyst was dried at 100 °C for 12 h and used in the subsequent experimental oxidative depolymerization step. The CeZrO_2_–CA catalyst's recyclability test for oxidative depolymerizing of extracted lignin at 160 °C for 30 min is shown in Fig. 16. As can be seen, after four successive recycling processes, vanillin production was practically steady. After the first recycling run, the vanillin production of the catalysts that had been previously used somewhat decreased. After four cycles, the yield decreased from 14.3% to 13.56%.

### Characterization of used catalyst

**Fig. 17:**
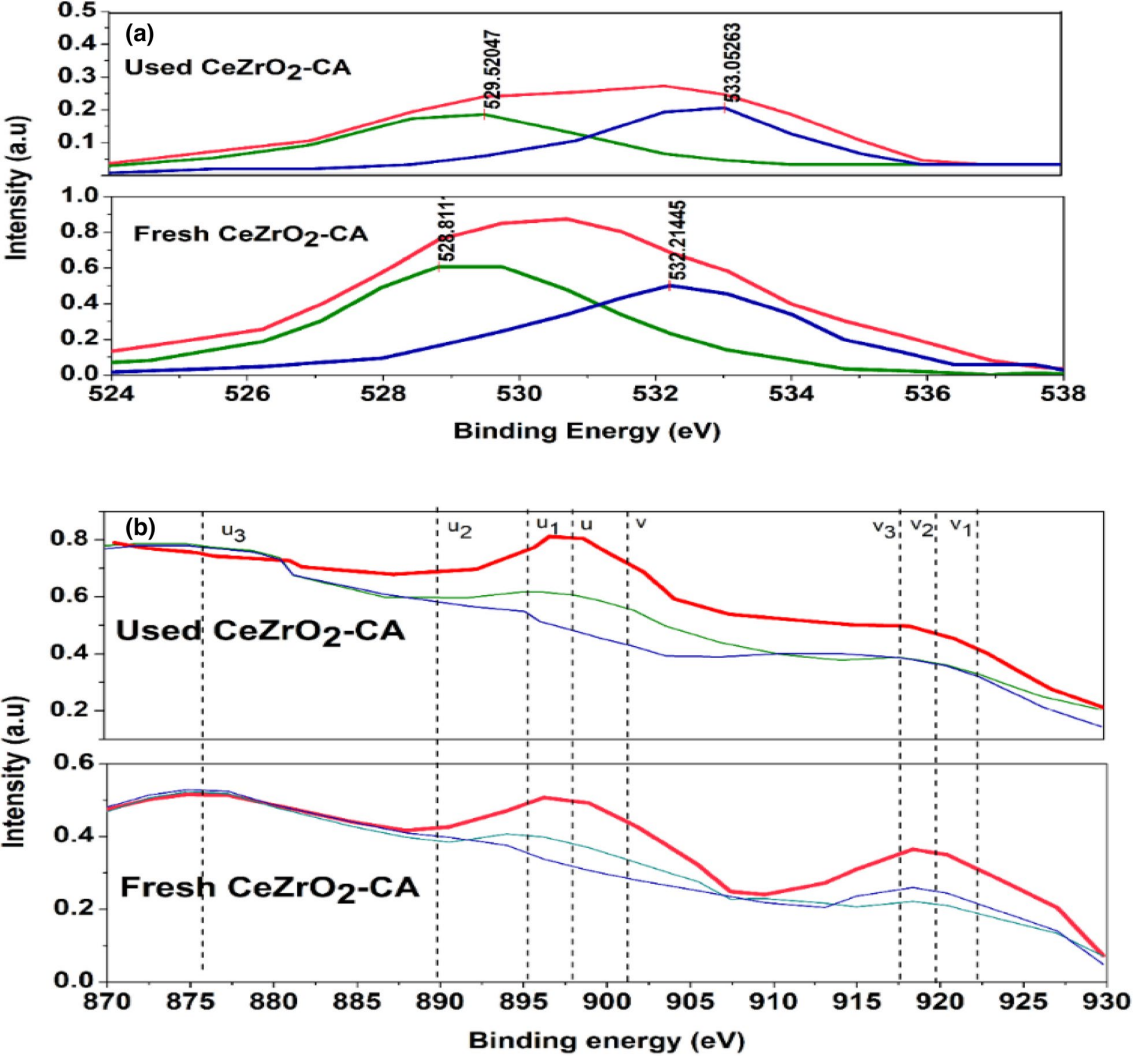
XPS spectra of (**a**) O 1 s and (**b**) Ce 3d of fresh and utilized CeZrO_2_–CA samples

The used CeZrO_2_–CA catalyst's surface properties were examined using X-ray photoelectron spectroscopy (XPS) to determine changes that occurred before and after the reaction. Figure 17 displays the XPS spectra of the Ce 3d and O 1 s areas. Both samples in Fig. 17a show a single peak that is divided into two overlapping sections. Surface-chemisorbed oxygen (O_α_) is represented by the greater energy spectrum 528.81 and529.52 eV, whereas lattice oxygen (O_β_) is represented by the lesser energy spectrum 532.2 and 533.05 eV for fresh and used catalyst, respectively. A comparison of the fresh CeZrO_2_–CA and the used CeZrO_2_–CA shows a decrease in the resulting signal intensities of the peaks linked to surface-bound oxygen (commonly O_α_), as shown in Fig. 17a. In addition, as shown in Table 4, the amounts of chemisorbed oxygen on the fresh CeZrO_2_–CA and the used CeZrO_2_–CA fell from 51.2% to 48.7%.

As shown in Fig. 17b, the 3d^10^4f^0^ state of Ce^4+^ was allocated to the peaks labeled v, v_2_, v_3_, u, u_2_, and u_3_, whereas the 3d^10^4f^1^ state of Ce^3+^ was assigned to the peaks labeled v_1_ and u_1_. It showed that Ce^4+^ was the predominant state, and Ce^3+^ was also present on the outermost layer of the two samples. According to Fig. 17b, the peak intensities representing Ce^4+^ reduced the following reaction when compared to fresh samples of CeZrO_2_–CA and utilized CeZrO_2_–CA. As shown in Table 5, the ratio of Ce^4+^ fell from 68.9% to 63.7%, respectively. It was discovered that the fresh catalyst has a greater capacity for adsorption and more chemically adsorbed oxygen on its surface, which encourages the oxidative depolymerization of lignin and produces more vanillin (Wang et al. 2023; Zhang et al. 2016; Zou et al. 2019).

**Table 5:** XPS data of fresh CeZrO_2_–CA and used CeZrO_2_–CA samples

Catalysts	Ce^4+^/(Ce^4+^ + Ce^3+^)	O_α_/(O_α_ + O_β_)
Fresh CeZrO_2_–CA	68.9%	51.2%
Used CeZrO_2_–CA	63.7%	48.7%

**Fig. 18:**
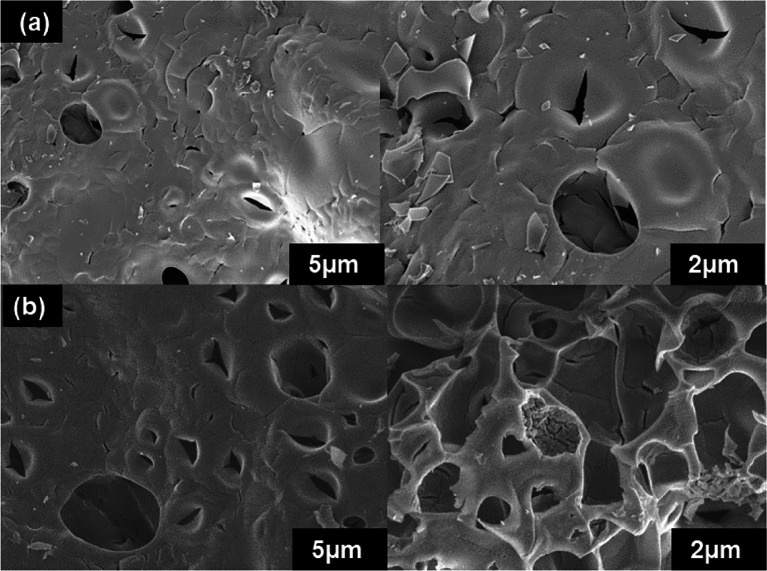
SEM analysis (**a**) fresh CeZrO_2_–CA, and (**b**) used CeZrO_2_–CA

The CeZrO_2_–CA used catalyst morphology before and after the oxidative depolymerization of lignin remains unchanged, as shown in Fig. 18a, b. The catalyst maintained its initial spongy shape, with holes or bubbles separating it. The absence of agglomeration particles for the used catalyst in Fig. 18b indicates that the production of Zr, Ce, and O agglomerates was leached throughout the process. The used CeZrO_2_–CA catalyst has a diameter between 27 and 28 nm.

**Fig. 19:**
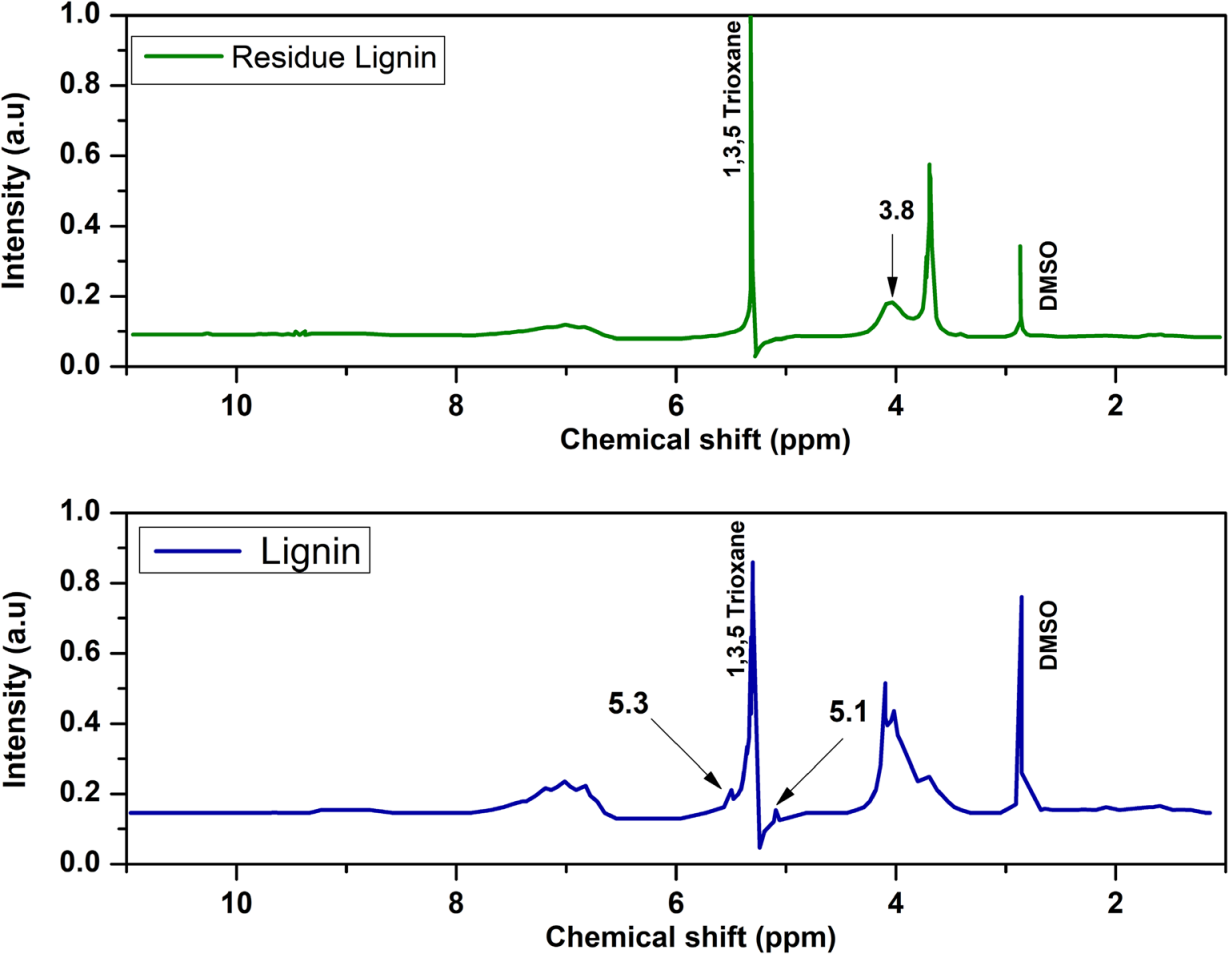
Proton NMR spectra of lignin and residue lignin

The proton NMR spectra of the residue and lignin utilizing the 1,3,5-trioxane internal standard are shown in Fig. 19. Despite having quantitative differences, the NMR spectra of lignin and lignin residue show a lot of similarities. The residue has a substantially higher amount of methoxy group than lignin, as evidenced by the peak at 3.8 ppm representing protons of the methoxy groups (–OCH3). According to studies, the presence of more methoxy groups in the residue indicates that the *β*-O-4 links will be successfully broken during the depolymerization event (Holding et al. 2018; Roy et al. 2021). The lignin and residue lignin both show distinct peak at 2.8 and 5.40 ppm due to DMSO and internal standard (1,3,5-trioxane), respectively. Again, the connections of *β*-o-4 and *β*-o-5 are represented by two minor peaks at 4.90 and 5.35 ppm, respectively (Assemat et al. 2018). The absence of the peak *β*-o-4 and *β*-5 in the residue lignin indicates that *β*-o-5 was successfully depolymerized to produce aromatic compounds (vanillin).

In addition, the sample spectra show broad peak ranges from 6.5 to 7.35 ppm attributed to the aromatic protons. Three minor peaks at 6.8, 6.96, and 7.12 ppm in this broad peak correspond to the guaiacol propane units found in the samples (Roy et al. 2021). Figure 19 demonstrates that the lignin is more aromatic than the residue, which suggests that the guaiacol unit was successfully broken down during the reaction. The spectra show that compared to lignin; the residue contains much more aliphatic proton but less aromatic proton. The greater aliphatic/aromatic ratio of the residue demonstrated that a significant portion of the lignin's aromatic components were reacted, providing more aliphatic components in the residue.

### Gas chromatography mass spectrometry (GCMS) analysis

**Fig. 20:**
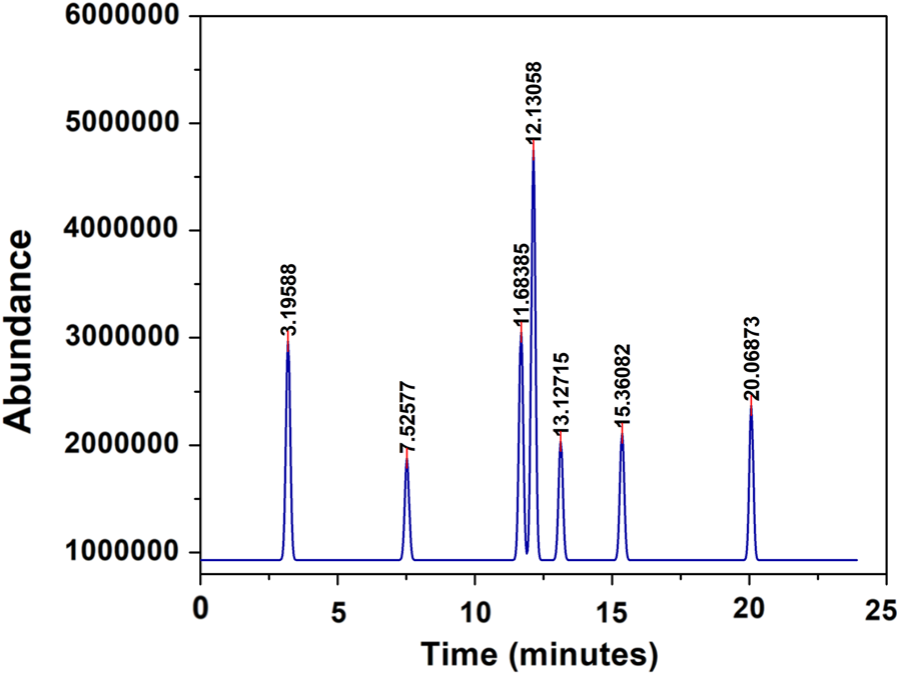
Typical GC/MS chromatogram of the kenaf stalk's degradation products

Due to the presence of aromatic compounds in the aqueous phase, GC–MS analysis revealed that CeZrO_2_–CA catalytic oxidation under optimal conditions (5wt% catalyst loading, 160 °C temperature, and 30 min) is a potential method for lignin depolymerization, which will primarily produce vanillin. The composition of wood products that have degraded is given in Table 6, and the GC chromatogram that shows the existence of the various composition products is derived from the direct oxidation of kenaf stalks and depicted in Fig. 20. In addition to the acetadol, guaiacol, 4-hydroxybenzaldehyde, vanillin, acetovanillone, and syringaldehyde are the main aromatic monomers from wood degradation included syringaldehyde and vanillin. Fortunately, vanillin, an aldehydic compound, demonstrated the biggest peaks in the chromatogram's region (30.15%) (Manassa and Seesuriyachan 2021; NIST02 2008).

**Table 6:** Retention time (RT), structure, and lignin-derived monomeric products detected by GC–MS analysis (NIST02 2008)

No.	RT (min)	Area (%)	Degradation product	Structure
1	3.19588	16.17023	Acetaldol	C_4_H_8_O_2_
2	7.52577	7.44059	Guaiacol	C_7_H_8_O_2_
3	11.68385	16.66506	4-Hydroxybenzaldehyde	C_7_H_6_O_2_
4	12.13058	30.1524	Vanillin	C_8_H_8_O_3_
5	13.12715	8.64944	Acetovanillone	C_9_H_10_O_3_
6	15.36082	9.25593	Syringaldehyde	C_9_H_10_O_4_

## Conclusion

In summary, we have used the citrate-complexation approach to successfully synthesize three catalysts: CeO_2_–CA, ZrO_2_–CA, and CeZrO_2_–CA. All the prepared catalysts have been fully analyzed using FTIR, XRD, TPO, FESEM, TEM, and BET to confirm the origin of their structural properties. Ultimately, the catalysts have been utilized for the direct oxidative depolymerization of kenaf stalks to generate vanillin. The catalytic activity was investigated, and vanillin has been produced. The effect of various reaction parameters such as reaction time, temperature, and catalyst dosage were investigated. Noteworthy, the CeZrO_2_–CA catalyst produced the highest vanillin yield of 9.90% for kenaf stalk using 5 wt% of CeZrO_2_–CA at an optimized reaction condition of 160 °C within 30 min. Furthermore, vanillin production using extracted lignin was studied employing CeZrO_2_–CA as a catalyst which yielded 14.3% vanillin. Finally, the catalysts show recyclability up to four times and stability which may be a potential catalyst for industrial applications.

## Data Availability

The article or supplemental material contains data.

## Abbreviations

Rpm: Revolutions per minute
Mbar: Millibar pressure
m^2^/g: Meter square per gram
cm^3^/g: Centimeter cube per gram
nm: Nanometers
mm: Milli meter
ºC: Degree Celsius
%: Percentage
wt%: Weight percentage
v/v: Volume/volume
h: Hour
min: Minute
CeO_2_: Cerium dioxide
EDXS: Energy dispersive X-ray spectroscopy
